# Opportunities and Challenges in Screening for Colorectal Cancer

**DOI:** 10.1089/pop.2023.0013

**Published:** 2023-08-14

**Authors:** Lesley-Ann Miller Wilson, Sara Browne, Jake Barnes, Nancy El Hoyek, Leah Helmueller, Maria Joao Janeiro, Barry Wendt

**Affiliations:** ^1^ Exact Sciences Corporation, Madison, Wisconsin, USA.; ^2^ St Elizabeth Healthcare, Edgewood, Kentucky, USA.; ^3^ Maple Health Group, LLC, New York, New York, USA.

**Keywords:** colorectal cancer, colonoscopy, guideline, multitarget stool DNA test, screening

## Abstract

Colorectal cancer (CRC) is a leading cause of mortality in the United States. Outcomes are greatly improved if CRC is detected early; hence, screening is currently recommended for adults aged 45 years and older at average risk for the disease. Despite this recommendation and the availability of accurate screening tests, the CRC screening rates are below those recommended. The goal of this study was to identify temporal trends (from 2015 to 2019) in CRC screening rates and the utilization of screening tests recommended for CRC detection among average-risk individuals within the St Elizabeth Healthcare system in Kentucky, United States. The primary population of interest was patients aged 50–75 years (the CRC screening was recommended for this age group at the time of the study). Deidentified data were sourced from patients' electronic health records, and the results showed that screening rates increased significantly from 26% in 2015 to 49% in 2019 (<0.0001). The incidence of any screening test also increased significantly from 2015 to 2019, for those who were due for screening (*P* < 0.05) and for the entire cohort (*P* < 0.1). The use of multitarget stool DNA (mt-sDNA) increased 40-fold over the study timeframe (*P* < 0.05). These study results confirm that CRC screening rates remain suboptimal, although incidence and adherence improved significantly in those aged 50–75 years from 2015 to 2019. The growing adoption and availability of mt-sDNA may be correlated with an increase in overall screening in this average-risk population.

## Introduction

Colorectal cancer (CRC) is the third most commonly diagnosed cancer and the second leading cause of cancer-related mortality in the United States.^1^ In 2022, the American Cancer Society (ACS) estimates there will be >151,030 new cases of CRC diagnosed and ∼52,580 deaths from CRC.^1^ However, survival rates are higher and outcomes are improved if CRC is detected at an early stage before cancer has spread, with the 5-year relative survival rate for patients diagnosed with localized CRC being 91% in comparison with 15% for those diagnosed with advanced-stage CRC.^2^

Importantly, screening tests to detect the disease in precancerous and early stages are widely available in the United States and have been shown to reduce CRC mortality.^3,4^ Hence, screening is strongly recommended (Grade A recommendation) by the United States Preventive Services Task Force (USPSTF) for adults aged 50–75 years at average risk of CRC, concluding that screening in this age group has a substantial net benefit. The most recent guidelines further recommend that CRC screening in average-risk individuals commences at the age of 45 years, as the incidence of both CRC and premalignant lesions increases sharply after this age.^5,6^

However, CRC screening rates are suboptimal and despite the USPSTF recommendations and the recognized benefits of screening, screening rates among people aged 50–75 years were reported to be 71.6% in 2020,^7^ which is below the National CRC Roundtable goal of 80% of the population screened in every community. To promote the uptake of CRC screening, a range of noninvasive tests are available and are recommended by the USPSTF, the ACS, and the National Comprehensive Cancer Network.

Recommended tests include a high-sensitivity guaiac fecal occult blood test (gFOBT), fecal immunochemical test (FIT), CT colonography (CTC), flexible sigmoidoscopy (SIG), and multitarget stool DNA (mt-sDNA). No 1 test is recommended over another, and the choice of test depends on physician and patient preference.^4,5^

The mt-sDNA test (Cologuard^®^) is the most recently endorsed CRC noninvasive screening test. The screening test was approved by the US Food and Drug Administration in August 2014 and added to the USPSTF guidelines in 2016 for individuals at average risk for CRC. The test is designed for the detection of colorectal neoplasia-associated DNA markers and for the presence of occult hemoglobin in human stool in adults 45 years and older who are at average risk for CRC. Based on the combined results of the DNA markers and hemoglobin, a qualitative “positive” or “negative” test result is provided.

Patients with positive results are required to complete a follow-up colonoscopy, whereas it is recommended that patients with negative results are subsequently rescreened at 3-year intervals.^5^ The test has been demonstrated to detect CRC with a high degree of sensitivity (92.3%) in a large clinical trial,^8^ although the uptake and impact of mt-sDNA in real-world settings have not yet been comprehensively explored. Understanding CRC screening patterns with the currently available screening tests may help providers devise strategies to improve screening rates, leading to better CRC detection and prevention.

Hence, the objectives of this study were 2-fold: the first was to identify temporal trends in CRC screening participation and CRC screening modality utilization use among average-risk, screen-eligible individuals in the St Elizabeth Healthcare system in Kentucky, United States, and the second was to assess the uptake of mt-sDNA within the system.

## Methods

### Study design

A retrospective medical record review of people aged 40 years and older at average risk for CRC, and eligible for screening within the St Elizabeth Healthcare system in Northern Kentucky, United States, was conducted. The primary population of interest was individuals aged 50–75 years old that was the recommended age range for CRC screening during the study period. Patients aged between 45 and 50 years and older than 75 years were also assessed separately in secondary analyses. Data for eligible patients who had a CRC screening test performed (any modality) between January 1, 2015 and December 31, 2019 were included. Study years were defined as 2015, 2016, 2017, 2018, and 2019 calendar years (ie, from January 1 to December 31 inclusive).

### Data sources

Data were sourced from the system's electronic health records. All data were deidentified in the extraction phase before being transferred as per the data transfer agreement. The study was approved by St. Elizabeth Institutional Review Board (IRB No. SEH 9/2018-032).

### Study population

Participants were eligible for inclusion in the study if they were aged 40 years or older at the date the CRC screening test was performed, their electronic medical record showed at least 1 visit within the previous 36 months to a provider within the system from 1 of the following specialties: family medicine, internal medicine, geriatrics, or obstetrics/gynecology. The primary analyses focused on individuals aged 50–75 years.

**FIG. 1.**
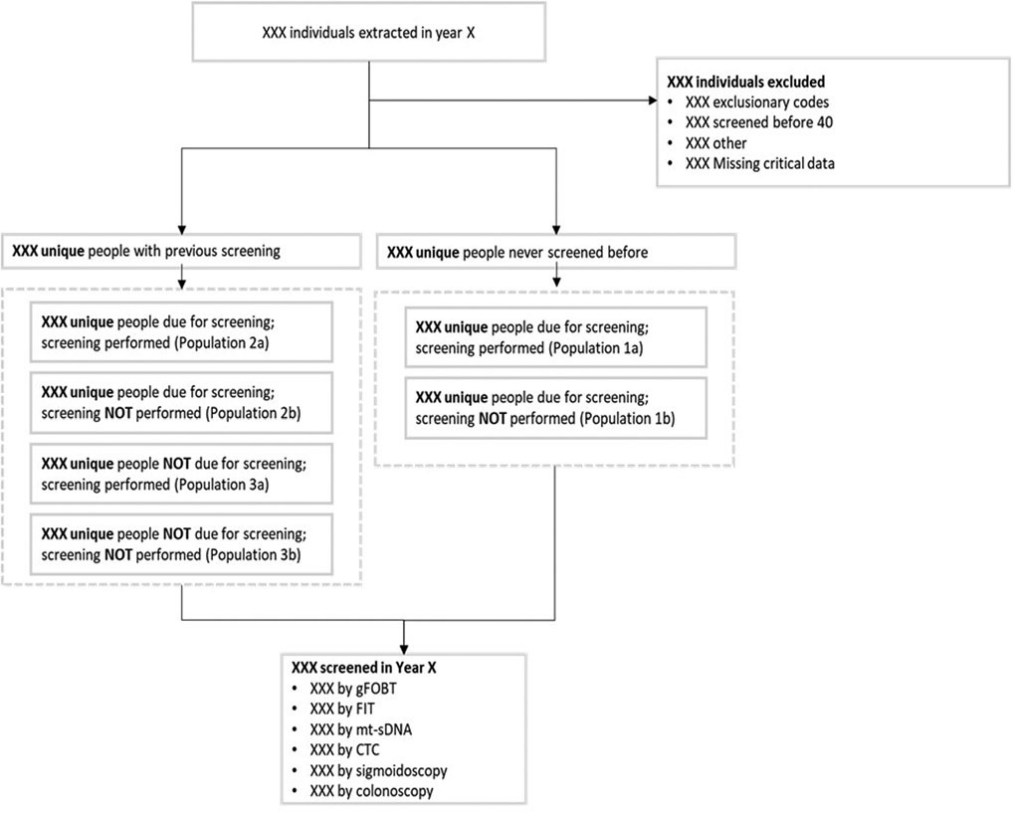
Study flow schema. CTC, CT colonography; FIT, fecal immunochemical test; gFOBT, guaiac fecal occult blood test; mt-sDNA, multitarget stool DNA.

Hospice patients were excluded from the study. Patients were also excluded if there was evidence of being above average risk for CRC before screening, as determined by the presence of at least 1 International Classification of Diseases (ICD)-9/ICD-10 code indicating the presence, history, or symptoms of benign or malignant colorectal neoplasms, colorectal polyps, inflammatory bowel disease (ulcerative colitis or Crohn's disease), a family history of CRC, familial adenomatous polyposis, or hereditary nonpolyposis CRC. The study flow schema is presented in Figure 1.

### Study measures and outcomes

Patient demographic information was extracted from the database including their age (as of December 31 of each study year), gender (male/female), ethnicity (American Indian/Pacific Islander, Asian, Black, White, Other), and Hispanic (yes/no). The primary outcome measures were screening adherence rates in the eligible population, and utilization of each CRC screening test (ie, test mix) for each study year. Screening adherence rates were calculated as the proportion of unique patients in the eligible population, who were up to date with CRC screening using any USPSTF-recommended screening test within each study year. The CRC screening test mix included gFOBT or FIT, mt-sDNA, CTC, SIG, and screening colonoscopy. Both outcomes were assessed for each study year from 2015 to 2019, inclusive.

### Statistical analyses

Descriptive statistics were calculated for continuous variables and included means and standard deviations, and frequencies for categorical variables. Screening adherence was summarized as counts and proportions, whereas the screening test mix was summarized as counts, proportion, “screening incidence” (newly screened within each measurement year per 1000 eligible population), and “screening rates” (those previously screened and newly screened within measurement year per 1000 eligible population).

Study outcomes were calculated for those aged 50–75 years, and secondary analyses were performed for the 2 other age groups (45 to 49 and >75 years). Trends in screening adherence over time (2015–2019) were assessed through binomial regression, and trends in screening incidences over time (2015–2019) for the overall cohort and across screening modalities were assessed using Gaussian regressions. All analyses were performed using R Statistical Software.^9^

## Results

A total of 195,122 unique individuals aged 50–75 years were included (Table 1). Across all 5 years (2015–2019), around 54% were female, >95% were White, and the majority were non-Hispanic (Table 2).

**Table 1.** Study Sample

	January 1, 2015–December 31, 2015		January 1, 2016–December 31, 2016		January 1, 2017–December 31, 2017		January 1, 2018–December 31, 2018		January 1, 2019–December 31, 2019	
No. of unique patients meeting the following criteria										
Total extracted	146,600		150,716		150,879		159,587		155,185	
Excluded due to exclusionary ICD-9 codes	11,254	7.7%	12,560	8.3%	14,229	9.4%	16,745	10.5%	19,512	12.6%
Excluded due to screening before age of 40 years	0	0.0%	0	0.0%	0	0.0%	0	0.0%	0	0.0%
Excluded due to missing critical data (ie, test dates)	0	0.0%	0	0.0%	0	0.0%	0	0.0%	0	0.0%
Excluded due to missing age or gender	9	0.0%	9	0.0%	5	0.0%	6	0.0%	2	0.0%
Total excluded	11,263	7.7%	12,569	8.3%	14,234	9.4%	16,751	10.5%	19,514	12.6%
Total remaining	124,074	84.6%	125,578	83.3%	122,411	81.1%	126,085	79.0%	116,157	74.9%
New patient	13,794	9.4%	13,245	8.8%	11,824	7.8%	11,939	7.5%	11,481	7.4%
Due for screening this year and performed it	959	7.0%	1014	7.7%	836	7.1%	709	5.9%	982	8.6%
Due for screening this year and did not perform it	12,160	88.2%	11,684	88.2%	10,652	90.1%	10,927	91.5%	10,194	88.8%
Not due for screening this year and performed it	30	0.2%	40	0.3%	17	0.1%	10	0.1%	8	0.1%
Not due for screening this year and did not perform it	645	4.7%	507	3.8%	319	2.7%	293	2.5%	297	2.6%
Existing patient	121,543	82.9%	124,902	82.9%	124,821	82.7%	130,897	82.0%	124,190	80.0%
Due for screening this year and performed it	6444	5.3%	7732	6.2%	9349	7.5%	8402	6.4%	11,392	9.2%
Due for screening this year and did not perform it	96,318	79.2%	94,481	75.6%	88,273	70.7%	89,614	68.5%	76,757	61.8%
Not due for screening this year and performed it	1593	1.3%	1866	1.5%	2392	1.9%	2075	1.6%	2321	1.9%
Not due for screening this year and did not perform it	17,188	14.1%	20,823	16.7%	24,807	19.9%	30,806	23.5%	33,720	27.2%

	January 1, 2015–December 31, 2015		January 1, 2016–December 31, 2016		January 1, 2017–December 31, 2017		January 1, 2018–December 31, 2018		January 1, 2019–December 31, 2019	
Screened this year (among final included sample)										
Screened this year by any modality	9026	7.3%	10,652	8.5%	12,594	10.3%	11,196	8.9%	14,703	12.7%
Screened this year by gFOBT	3400	2.7%	3498	2.8%	3167	2.6%	575	0.5%	360	0.3%
Screened this year by FIT	500	0.4%	513	0.4%	1118	0.9%	1875	1.5%	1056	0.9%
Screened this year by mt-sDNA	182	0.1%	593	0.5%	1461	1.2%	2302	1.8%	7057	6.1%
Screened this year by CTC	0	0.0%	0	0.0%	0	0.0%	0	0.0%	0	0.0%
Screened this year by sigmoidoscopy	50	0.0%	65	0.1%	65	0.1%	79	0.1%	54	0.0%
Screened this year by colonoscopy	4894	3.9%	5983	4.8%	6783	5.5%	6365	5.0%	6176	5.3%

CTC, CT colonography; FIT, fecal immunochemical test; gFOBT, guaiac fecal occult blood test; ICD, International Classification of Diseases; mt-sDNA, multitarget stool DNA.

**Table 2.** Demographic Characteristics for Individuals Aged 50–75 years

	2015		2016		2017		2018		2019	
	N	%	N	%	N	%	N	%	N	%
Total	83,746	100.00	85,903	100.00	85,066	100.00	88,788	100.00	85,171	100.00
Gender										
Females	44,290	52.89	45,382	52.83	44,994	52.89	47,027	52.97	45,447	53.36
Males	39,456	47.11	40,521	47.17	40,072	47.11	41,761	47.03	39,724	46.64
Race										
American Indian/Pacific Islander	121	0.14	132	0.15	139	0.16	147	0.17	144	0.17
Asian	535	0.64	591	0.69	629	0.74	678	0.76	652	0.77
Black	1490	1.78	1559	1.81	1575	1.85	1710	1.93	1626	1.91
White	80,141	95.70	82,091	95.56	81,326	95.60	84,688	95.38	81,292	95.45
Other	1459	1.74	1530	1.78	1397	1.64	1565	1.76	1457	1.71
Hispanic										
Yes	710	0.85	783	0.91	799	0.94	894	1.01	843	0.99
No	83,036	99.15	85,120	99.09	84,267	99.06	87,894	98.99	84,328	99.01
Payment type										
Commercial	100	0.12	297	0.35	819	0.96	1822	2.05	4788	5.62
Medicare	218	0.26	469	0.55	1044	1.23	1992	2.24	3722	4.37
Medicaid	4	0.00	19	0.02	71	0.08	87	0.10	111	0.13
Self-Pay	1	0.00	2	0.00	6	0.01	6	0.01	9	0.01
Other/unknown	83,423	100	85,116	99	83,126	98	84,879	96	76,539	90

### CRC screening adherence

**FIG. 2.**
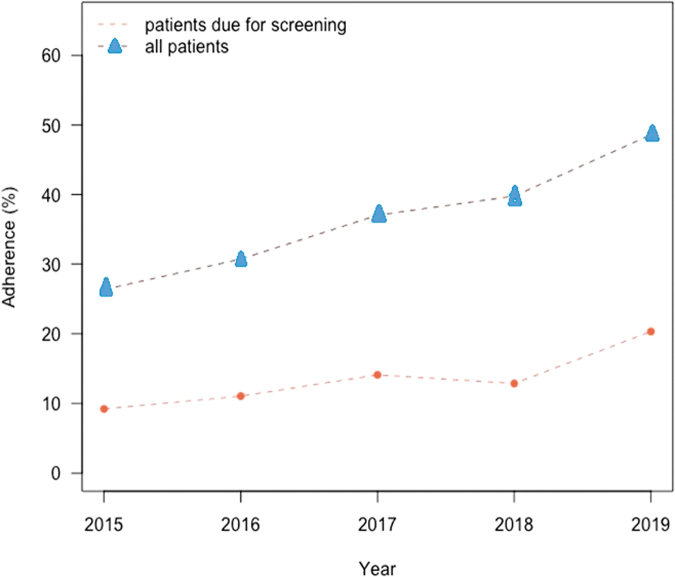
Screening adherence for patients aged 50–75 years.

Screening adherence rates are presented in Figure 2, which shows that overall screening adherence rates increased from 26% (22,073 patients) in 2015 to 49% (41,378 patients) in 2019, representing a significant increase over the 5-year study period (*P* < 0.0001). There was a similar significant increase in adherence rates for patients who were due for screening, increasing from 9% (6256 patients) in 2015 to 20% (11,174 patients) in 2019 (*P* < 0.0001).

### CRC screening test mix

CRC screening test mix results are summarized in Table 3 that shows that the incidence of any screening modality increased significantly from 2015 to 2019, both for those who were due for screening (*P* < 0.05) and for the whole cohort (*P* < 0.1). However, the utilization of specific tests changed considerably over the study timeframe with the use of gFOBT, and FIT decreasing significantly from 2015 to 2019 (68.5%), declining from 38.68 per 1000 persons in 2015 to 12.19 tests per 1000 persons in 2019 (*P* < 0.1). By comparison, there was a 40-fold increase in the use of mt-sDNA, with the screening incidence increasing from 1.99 in 2015 to 79.55 tests per 1000 persons in 2019 (*P* < 0.05).

**Table 3.** Screening Test Mix—Screening Counts, Proportions Incidence, and Rates for Individuals Aged 50–75 years

Test	Counts per year							Proportion (%) per year						
	2015	2016	2017	2018	2019	Linear trend		2015	2016	2017	2018	2019	Linear trend	
						*p*	Direction						*p*	Direction
Any screening modality	7637	9003	10,865	9607	13,153	<0.0001	Positive							
High sensitivity gFOBT	2953	3009	2809	452	271	<0.001	Negative	38.67	33.42	25.85	4.70	2.06	<0.0001	Negative
FIT	287	304	858	1562	767			3.76	3.38	7.90	16.26	5.83		
mt-sDNA	167	541	1367	2170	6775	<0.001	Positive	2.19	6.01	12.58	22.59	51.51	<0.0001	Positive
Screening colonoscopy	4200	5110	5794	5373	5304	<0.001	Positive	55.00	56.76	53.33	55.93	40.33	<0.0001	Negative
Sigmoidoscopy	30	39	37	50	36	>0.1	N/A	0.39	0.43	0.34	0.52	0.27	>0.1	N/A

Test	Screening incidence for those due, per 1000							Screening incidence for all, per 1000						
	2015	2016	2017	2018	2019	Linear trend		2015	2016	2017	2018	2019	Linear trend	
						*p*	Direction						*p*	Direction
Any screening modality	92.10	110.51	141.04	128.63	203.29	<0.05	Positive	91.19	104.80	127.72	108.20	154.43	<0.1	Positive
High sensitivity gFOBT	33.40	34.23	33.13	4.45	2.38	>0.1	N/A	35.26	35.03	33.02	5.09	3.18	<0.1	Negative
FIT	3.03	3.42	11.21	21.95	10.81			3.43	3.54	10.09	17.59	9.01		
mt-sDNA	2.38	7.71	21.18	33.73	118.53	<0.1	Positive	1.99	6.30	16.07	24.44	79.55	<0.1	
Screening colonoscopy	53.04	64.83	75.26	68.20	71.32	>0.1	N/A	50.15	59.49	68.11	60.51	62.27	>0.1	N/A
Sigmoidoscopy	0.24	0.31	0.26	0.29	0.25	>0.1	N/A	0.36	0.45	0.43	0.56	0.42	>0.1	N/A

Test	Screening rates for those who performed a test, per 1000							Screening rates for all, per 1000						
	2015	2016	2017	2018	2019	Linear trend		2015	2016	2017	2018	2019	Linear trend	
						*p*	Direction						*p*	Direction
Any screening modality	91.19	104.80	127.72	108.20	154.43	<0.1	Positive	875.17	869.67	881.05	871.07	906.59	>0.1	N/A
High sensitivity gFOBT	35.26	35.03	33.02	5.09	3.18	<0.1	Negative	258.22	201.14	155.92	74.08	51.19	0.001	Negative
FIT	3.43	3.54	10.09	17.59	9.01			25.69	21.42	38.11	56.88	30.30		
mt-sDNA	1.99	6.30	16.07	24.44	79.55	<0.1		12.02	33.51	71.41	115.05	245.02	0.007	Positive
Screening colonoscopy	50.15	59.49	68.11	60.51	62.27	>0.1	N/A	574.72	609.01	611.91	621.07	576.97	>0.1	N/A
Sigmoidoscopy	0.36	0.45	0.43	0.56	0.42	>0.1	N/A	4.53	4.59	3.70	4.00	3.11	<0.05	Negative

FIT, fecal immunochemical test; gFOBT, guaiac fecal occult blood test; mt-sDNA, multitarget stool DNA.

Otherwise, the screening incidence of colonoscopy remained almost constant during the same period, with an average of 60.11 tests per 1000 persons per year from 2015 to 2019. Screening rates for ages 50–75 years for each study year are presented in Table 2, which shows a pattern similar to that of incidence rates. From 2015 to 2019, overall screening rates increased significantly for any screening modality from 875.2 per 1000 persons to 906.6 per 1000 (*P* > 0.1); use of gFOBT and FIT declined significantly (*P* = 0.001), whereas the use of mt-sDNA increased significantly (*P* = 0.007) and colonoscopy use remained about the same. Similar patterns were evident for those who underwent a screening test.

### CRC screening utilization among younger (<50 years old) and older (75+) populations

Data regarding the proportions of patients of all ages who were adherent (those due for a test) to screening recommendations are displayed in Supplementary Figure S1. By comparison with the group aged 50–75 years, adherence was lower for patients due for a test in the other age groups (45–49 years, ≥76 years) and was <10% for each study year. The rate declined significantly from 2015 to 2019 for those due for a test in the 45–49 years age group (1.8% in 2015, 1.6% in 2019 [*P* < 0.05]). By comparison, the rate increased significantly for patients aged >75 years who were due for a test from 2015 to 2019 (4.1% in 2015, 5.7% in 2019 [*P* < 0.001]).

Patterns of screening test mix also differed by age group with the younger age group demonstrating a significantly decreased use of gFOBT tests (*P* < 0.001) and no change in utilization of mt-sDNA tests or colonoscopies. Otherwise, a significant decrease in the use of gFOBT tests was apparent (*P* < 0.001) in the oldest age group (≥76 years), together with a corresponding increase in mt-sDNA testing (*P* < 0.001) and colonoscopies (*P* < 0.001).

A different pattern was demonstrated for overall adherence rates. Although overall adherence in the group aged 45–49 years increased from 6.0% in 2015 to 7.8% in 2019 (*P* < 0.001), the oldest age group (≥76 years) increased from 18.4% in 2015 to 31.0% in 2019 (*P* < 0.001). These data are available in the Supplementary Data.

## Discussion

Findings from this study show that adherence to recommended screening for CRC improved significantly from 2015 to 2019 in this population of adults aged 50–75 years at average risk for CRC. Although the overall rate almost doubled (increasing from 26% to 49%) and more than doubled in those due for screening (increasing from 9% to 20%), screening rates remained considerably lower in 2019 than the 80% target established by the National CRC Roundtable.^10,11^ Significant changes in the types of tests used were also observed. Over the 5 years, the use of gFOBT and FIT declined sharply while there was a 40-fold increase in the use of mt-sDNA over the same time, with colonoscopy rates essentially remaining unchanged.

A similar pattern was evident in patients aged 76 years and older across the 5 years, although the increase in screening rates was less pronounced, increasing from 4.1% to 5.7% in those due for a test, and from 18.4% to 31.0% overall. By comparison, screening rate in the younger age group (45–49 year-olds) was the lowest (<10% each year) and declined in those due for a test from 2015 to 2019. Future studies are needed to understand reasons for these interesting patterns among younger than 50 and older than 75-year-old individuals. These results indicate that there is a need to achieve higher rates of CRC screening, perhaps by adopting noninvasive screening test options that can be performed at home.^12,13^

Findings from this study are generally consistent with those of another recently published retrospective study of electronic health care records examining CRC screening behaviors and patterns among patients aged ≥40 years in the Ascension Wisconsin health system from 2015 to 2018.^12^ Data for 172,045 unique patients were included in the analysis and results showed that overall adherence increased significantly over the 4 years of the study, from 42,928 to 59,593 adults or 35.6% to 46.6% (*P* < 0.0001). Utilization of the types of screening tests also changed over the study's timeframe with the use of gFOBT and FIT demonstrating a 1.2-fold increase and mt-sDNA increasing 4.6-fold while colonoscopies decreased by 8%.

The greater availability and adoption of mt-sDNA testing may have been associated with the overall increase in screening. Another longitudinal cohort study using administrative claims data to examine CRC screening use also reported similar patterns.^13^ In this study, the use of gFOBT decreased between 2011 (1088 of 6241 eligible individuals [17.7%]) and 2019 (195 of 2943 eligible individuals [6.6%]), and the use of mt-sDNA increased between 2016 (58 of 3014 eligible individuals [1.9%]) and 2019 (418 of 2943 eligible individuals [14.2%]). Trends in FIT or screening colonoscopy were not consistent.

Of note, the St. Elizabeth health system decided to adopt and promote utilization of mt-sDNA as an option for CRC screening. Other screening options included colonoscopy and FIT. Observed barriers to improving colonoscopy uptake were costs, loss of time or income from work for patients and caregivers, transportation, reluctance to complete the bowel prep required for colonoscopy, anesthesia risk, complication risk such as perforation, and access to screening colonoscopies. FIT testing was felt to be the least sensitive test and required annual follow-up. For FIT testing, a labor-intensive process would need to be in place to complete the annual rescreen. Hence, the administrators at the health system pivoted to using the mt-sDNA test to help improve screening rates.

Results from this study also indicated that the greatest increase in mt-sDNA use was from 2018 to 2019, when its use tripled. This steep increase may have been due to several quality improvement projects and screening enhancement initiatives that occurred within the St Elizabeth Health System at that time. One such initiative was the implementation of shared decision making between providers and patients allowing patients to participate in the decision-making process and being able to choose their preferred screening test. Several operational changes also occurred in 2018 and 2019 to streamline processes around the ordering of the mt-sDNA test.

These included an HL-7 implementation, Smart Set CRC ordering, and quality dashboard upgrades. Finally, there was a reduction in the gap payment patients were required to pay for the mt-sDNA test as a consequence of the introduction of a bulk ordering strategy for patients due for CRC screening. Although we cannot say with certainty which of these strategies had the greatest impact, overall, they appear to have been effective in significantly increasing screening rates over the 2 years. A systematic literature review including studies from January 1, 1996 to December 17, 2017 showed that multilevel interventions such as mailed FIT outreach, reminders, and provider alerts implemented widely across the health systems to promote high-quality screening programs were effective in improving CRC screening rates.^14^

It is also important to note that a positive mt-sDNA test should be followed by a colonoscopy to confirm the findings and complete the screening paradigm. Future studies should focus on understanding colonoscopy completion rates after positive mt-sDNA results.

Although we have reported recent data regarding CRC screening rates and trends over the 5 years from 2015 to 2019 using a relatively large data set, the study is not without limitations. As the data have been sourced from 1 health care system within a single US state, it is unclear how generalizable these findings are to other US states and populations. There are also potential limitations relating to the use of administrative data, including possible inaccuracies within the data. For example, a test may have been incorrectly coded as a screening test when it was for diagnostic purposes or the reverse, leading to an over or underestimation of CRC adherence rates.

In addition, the system would not have included a screening test if a test had been purchased privately, and although this is not likely to have been a frequent occurrence, it would nevertheless lead to an underestimation of screening rates.

## Conclusions

In this retrospective study of average-risk individuals aged 50–75 years old, adherence to CRC screening increased significantly from 26% in 2015 to 49% in 2019. Moreover, among those who were up to date with their CRC screening, there was a substantial increase in the proportion of individuals receiving an mt-sDNA screening test from 2015 to 2019. Results from this study suggest that the growing adoption of mt-sDNA may be correlated with an increase in overall screening in this average-risk population, which is consistent with the results of previous studies.

These results are important for providers and health systems who are looking to optimize strategies to improve screening rates among an average-risk population. Strategies such as adopting use of at-home stool-based screening options may boost CRC screening rates. Further research is critical to corroborate the findings suggested by this single health system survey.

## Supplementary Material

Supplemental data
