# Supplementary material for: Opportunities and Challenges in Screening for Colorectal Cancer
Source: Popul Health Manag. 2023 Aug 14;26(4):246–53. doi: 10.1089/pop.2023.0013 (PMC10457624; doi:10.1089/pop.2023.0013)
Supplement: Supplemental data [file Supp_DataS1.docx]

**Supplementary Figures and Tables**

**Supplementary Figure 1. Adherence rates for those due for testing: all ages**


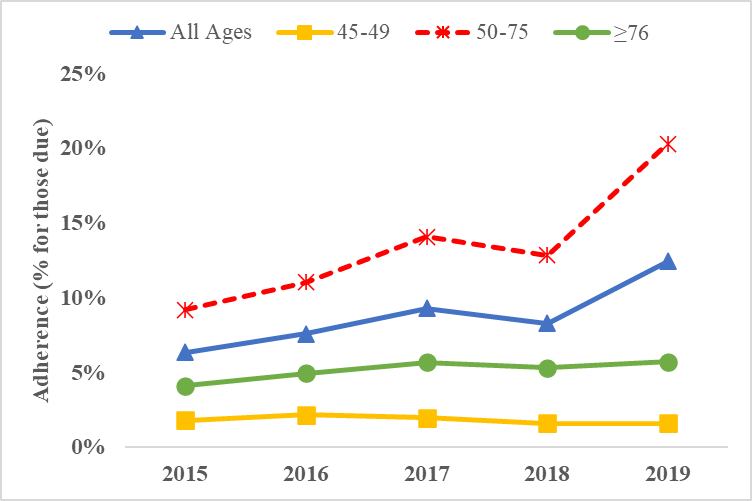


**Supplementary Table 1: Demographic characteristics for individuals (all ages)**

|  | **2015** | | **2016** | | **2017** | | **2018** | | **2019** | |
| --- | --- | --- | --- | --- | --- | --- | --- | --- | --- | --- |
|  | **N** | **%** | **N** | **%** | **N** | **%** | **N** | **%** | **N** | **%** |
| **Total** | 135,337 | 100.00% | 138,147 | 100.00% | 136,645 | 100.00% | 142,836 | 100.00% | 135,671 | 100.00% |
| **Age as of Dec 31st** |  |  |  |  |  |  |  |  |  |  |
| 40-44 | 15,813 | 11.68% | 15,891 | 11.50% | 15,770 | 11.54% | 17,033 | 11.92% | 15,767 | 11.62% |
| 45-49 | 19,900 | 14.70% | 20,105 | 14.55% | 19,486 | 14.26% | 19,916 | 13.94% | 17,959 | 13.24% |
| 50-75 | 83,746 | 61.88% | 85,903 | 62.18% | 85,066 | 62.25% | 88,788 | 62.16% | 85,171 | 62.78% |
| ≥76 | 15,878 | 11.73% | 16,248 | 11.76% | 16,323 | 11.95% | 17,099 | 11.97% | 16,774 | 12.36% |
| **Sex** |  |  |  |  |  |  |  |  |  |  |
| Females | 72,607 | 53.65% | 74,056 | 53.61% | 73,329 | 53.66% | 76,814 | 53.78% | 73,681 | 54.31% |
| Males | 62,730 | 46.35% | 64,091 | 46.39% | 63,316 | 46.34% | 66,022 | 46.22% | 61,990 | 45.69% |
| **Race** |  |  |  |  |  |  |  |  |  |  |
| American Indian/Pacific Islander | 198 | 0.15% | 208 | 0.15% | 220 | 0.16% | 243 | 0.17% | 233 | 0.17% |
| Asian | 1,049 | 0.78% | 1,176 | 0.85% | 1,212 | 0.89% | 1,305 | 0.91% | 1,176 | 0.87% |
| Black | 2,458 | 1.82% | 2,613 | 1.89% | 2,655 | 1.94% | 2,898 | 2.03% | 2,738 | 2.02% |
| White | 128,741 | 95.13% | 131,107 | 94.90% | 129,671 | 94.90% | 135,199 | 94.65% | 128,654 | 94.83% |
| Other | 2,891 | 2.14% | 3,043 | 2.20% | 2,887 | 2.11% | 3,191 | 2.23% | 2,870 | 2.12% |
| **Hispanic** |  |  |  |  |  |  |  |  |  |  |
| Yes | 1,395 | 1.03% | 1,537 | 1.11% | 1,564 | 1.14% | 1,687 | 1.18% | 1,583 | 1.17% |
| No | 133,942 | 98.97% | 136,610 | 98.89% | 135,081 | 98.86% | 141,149 | 98.82% | 134,088 | 98.83% |
| **Payment type** |  |  |  |  |  |  |  |  |  |  |
| Commercial | 111 | 0.08% | 311 | 0.23% | 847 | 0.62% | 1,867 | 1.31% | 4,828 | 3.56% |
| Medicare | 262 | 0.19% | 576 | 0.42% | 1,247 | 0.91% | 2,385 | 1.67% | 4,345 | 3.20% |
| Medicaid | 5 | 0.00% | 20 | 0.01% | 71 | 0.05% | 87 | 0.06% | 111 | 0.08% |
| Self Pay | 1 | 0.00% | 2 | 0.00% | 6 | 0.00% | 6 | 0.00% | 9 | 0.01% |
| Other/unknown | 134,958 | 99.7% | 137,238 | 99.3% | 134,474 | 98.4% | 138,488 | 97.0% | 126,375 | 93.1% |

**Supplementary Table 2: Screening Test Mix – Screening counts, proportions incidence, and rates for individuals aged 50-75**

|  | **Counts** | | | | | | | **Proportion (%)** | | | | | | |
| --- | --- | --- | --- | --- | --- | --- | --- | --- | --- | --- | --- | --- | --- | --- |
|  | **2015** | **2016** | **2017** | **2018** | **2019** | **Linear trend** | | **2015** | **2016** | **2017** | **2018** | **2019** | **Linear trend** | |
|  |  |  |  |  |  | **p-value** | **direction** |  |  |  |  |  | **p-value** | **direction** |
| **Overall** |  |  |  |  |  |  |  |  |  |  |  |  |  |  |
| any screening modality | **9,026** | **10,652** | **12,594** | **11,196** | **14,703** | 0.002 | positive |  |  |  |  |  |  |  |
| high sensitivity gFOBT (gFOBT) | 3,400 | 3,498 | 3,167 | 575 | 360 | 0.002 | negative | 37.67% | 32.8% | 25.15% | 5.14% | 2.45% | 0.002 | negative |
| FIT | 500 | 513 | 1,118 | 1,875 | 1,056 |  |  | 5.54% | 4.8% | 8.88% | 16.75% | 7.18% |  |  |
| mt-sDNA | 182 | 593 | 1,461 | 2,302 | 7,057 | 0.002 | positive | 2.02% | 5.6% | 11.60% | 20.56% | 48.00% | 0.002 | positive |
| screening Colonoscopy | 4,894 | 5,983 | 6,783 | 6,365 | 6,176 | 0.002 | positive | 54.22% | 56.2% | 53.86% | 56.85% | 42.01% | 0.002 | negative |
| sigmoidoscopy | 50 | 65 | 65 | 79 | 54 | >0.1 | N/A | 0.55% | 0.6% | 0.52% | 0.71% | 0.37% | <0.1 | negative |
| **Aged as of Dec 31st:**  **45 to 49** |  |  |  |  |  |  |  |  |  |  |  |  |  |  |
| any screening modality | **384** | **463** | **427** | **367** | **319** | 0.003 | negative |  |  |  |  |  |  |  |
| high sensitivity gFOBT (gFOBT) | 116 | 135 | 80 | 31 | 12 | 0.002 | negative | 30.21% | 29.2% | 18.74% | 8.45% | 3.76% | 0.002 | negative |
| FIT | 28 | 28 | 28 | 43 | 41 |  |  | 7.29% | 6.0% | 6.56% | 11.72% | 12.85% |  |  |
| mt-sDNA | 0 | 1 | 0 | 0 | 0 | N/A | | 0.00% | 0.2% | 0.00% | 0.00% | 0.00% | N/A | |
| screening Colonoscopy | 232 | 295 | 317 | 290 | 266 | >0.1 | N/A | 60.42% | 63.7% | 74.24% | 79.02% | 83.39% | 0.001 | positive |
| sigmoidoscopy | 8 | 4 | 2 | 3 | 0 | 0.006 | negative | 2.08% | 0.9% | 0.47% | 0.82% | 0.00% | <0.05 | negative |

| **Aged as of Dec 31st: ≥76** |  | |  | |  | |  |  | |  | |  | | |  |  | |  | |  | |  | | |  |  | |  |
| --- | --- | --- | --- | --- | --- | --- | --- | --- | --- | --- | --- | --- | --- | --- | --- | --- | --- | --- | --- | --- | --- | --- | --- | --- | --- | --- | --- | --- |
| any screening modality | **752** | | **901** | | **1,006** | | **981** | **1,001** | | 0.002 | | positive | | |  |  | |  | |  | |  | | |  |  | |  |
| high sensitivity gFOBT (gFOBT) | 273 | | 277 | | 228 | | 70 | 66 | | <0.002 | | negative | | | 36.30% | 30.7% | | 22.66% | | 7.14% | | 6.59% | | | <0.002 | negative | |  |
| FIT | 166 | | 169 | | 213 | | 241 | 232 | |  |  |  |  |  | 22.07% | 18.8% | | 21.17% | | 24.57% | | 23.18% | | |  |  |  |  |
| mt-sDNA | 15 | | 51 | | 94 | | 132 | 281 | | >0.002 | | positive | | | 1.99% | 5.7% | | 9.34% | | 13.46% | | 28.07% | | | <0.002 | positive | |  |
| screening Colonoscopy | 291 | | 387 | | 451 | | 518 | 405 | | 0.002 | | positive | | | 38.70% | 43.0% | | 44.83% | | 52.80% | | 40.46% | | | <0.05 | positive | |  |
| sigmoidoscopy | 7 | | 17 | | 20 | | 20 | 17 | | <0.1 | | positive | | | 0.93% | 1.9% | | 1.99% | | 2.04% | | 1.70% | | | >0.1 | N/A | |  |
|  | **Screening Incidence** | | | | | | | | | | | | | | | | | | | | | | | | | | | |
|  | **Screening incidence for those due, per 1000** | | | | | | | | | | | | | **Screening incidence for all, per 1000** | | | | | | | | | | | | | | |
|  | **2015** | **2016** | | **2017** | | **2018** | | | **2019** | | **Linear trend** | | | **2015** | | | **2016** | | **2017** | | **2018** | | **2019** | **Linear trend** | | | | |
|  |  |  |  |  |  |  |  |  |  |  | **p-value** | | **direction** |  |  |  |  |  |  |  |  |  |  | **p-value** | | | **direction** | |
| **Overall** |  |  | |  | |  | | |  | |  | |  |  | | |  | |  | |  | |  |  | | |  | |
| any screening modality | **63.88** | **76.11** | | **93.35** | | **83.09** | | | **124.58** | | <0.05 | | positive | **66.69** | | | **77.11** | | **92.17** | | **78.38** | | **108.37** | <0.1 | | | positive | |
| high sensitivity gFOBT (gFOBT) | 22.81 | 23.49 | | 21.67 | | 3.35 | | | 1.97 | | >0.1 | | N/A | 25.12 | | | 25.32 | | 23.18 | | 4.03 | | 2.65 | <0.1 | | | negative | |
| FIT | 3.29 | 3.42 | | 8.26 | | 14.40 | | | 8.23 | |  |  |  | 3.69 | | | 3.71 | | 8.18 | | 13.13 | | 7.78 |  |  |  |  |  |
| mt-sDNA | 1.50 | 4.92 | | 12.86 | | 19.94 | | | 68.08 | | <0.1 | | positive | 1.34 | | | 4.29 | | 10.69 | | 16.12 | | 52.02 | <0.1 | | | positive | |
| screening Colonoscopy | 36.04 | 43.97 | | 50.24 | | 45.10 | | | 46.06 | | >0.1 | | N/A | 36.16 | | | 43.31 | | 49.64 | | 44.56 | | 45.52 | >0.1 | | | N/A | |
| sigmoidoscopy | 0.25 | 0.31 | | 0.32 | | 0.30 | | | 0.24 | | >0.1 | | N/A | 0.37 | | | 0.47 | | 0.48 | | 0.55 | | 0.40 | >0.1 | | | N/A | |

| **Aged as of Dec 31st:**  **45 to 49** |  |  |  |  |  |  |  |  |  | |  | |  | |  | |  | |  |
| --- | --- | --- | --- | --- | --- | --- | --- | --- | --- | --- | --- | --- | --- | --- | --- | --- | --- | --- | --- |
| any screening modality | **18.10** | **21.80** | **19.98** | **16.26** | **16.87** | >0.1 | N/A | **19.30** | **23.03** | | **21.91** | | **18.43** | | **17.76** | | >0.1 | | N/A |
| high sensitivity gFOBT (gFOBT) | 5.72 | 6.83 | 4.01 | 1.49 | 0.71 | <0.05 | negative | 5.83 | 6.71 | | 4.11 | | 1.56 | | 0.67 | | <0.05 | | negative |
| FIT | 1.36 | 1.10 | 1.35 | 1.92 | 2.32 |  |  | 1.41 | 1.39 | | 1.44 | | 2.16 | | 2.28 | |  |  |  |
| mt-sDNA | 0.00 | 0.05 | 0.00 | 0.00 | 0.00 | N/A | | 0.00 | 0.05 | | 0.00 | | 0.00 | | 0.00 | | N/A | | |
| screening Colonoscopy | 10.81 | 13.77 | 14.57 | 12.74 | 13.84 | >0.1 | N/A | 11.66 | 14.67 | | 16.27 | | 14.56 | | 14.81 | | >0.1 | | N/A |
| sigmoidoscopy | 0.21 | 0.05 | 0.05 | 0.11 | 0.00 | >0.1 | N/A | 0.40 | 0.20 | | 0.10 | | 0.15 | | 0.00 | | <0.05 | | negative |
| **Aged as of Dec 31st: ≥76** |  |  |  |  |  |  |  |  |  | |  | |  | |  | |  | |  |
| any screening modality | **41.35** | **49.77** | **57.02** | **53.20** | **57.24** | <0.1 | positive | **47.36** | **55.45** | | **61.63** | | **57.37** | | **59.68** | | >0.1 | | N/A |
| high sensitivity gFOBT (gFOBT) | 15.39 | 15.10 | 13.49 | 3.60 | 3.42 | <0.1 | negative | 17.19 | 17.05 | | 13.97 | | 4.09 | | 3.93 | | <0.05 | | negative |
| FIT | 9.62 | 10.04 | 12.26 | 13.09 | 13.68 |  |  | 10.45 | 10.40 | | 13.05 | | 14.09 | | 13.83 | |  |  |  |
| mt-sDNA | 0.89 | 3.57 | 6.28 | 8.96 | 20.11 | <0.05 | positive | 0.94 | 3.14 | | 5.76 | | 7.72 | | 16.75 | | <0.05 | | positive |
| screening Colonoscopy | 15.16 | 20.31 | 23.99 | 26.95 | 19.30 | >0.1 | N/A | 18.33 | 23.82 | | 27.63 | | 30.29 | | 24.14 | | >0.1 | | N/A |
| sigmoidoscopy | 0.30 | 0.74 | 1.00 | 0.61 | 0.73 | >0.1 | N/A | 0.44 | 1.05 | | 1.23 | | 1.17 | | 1.01 | | >0.1 | | N/A |
|  | **Screening Rates** | | | | | | | | | | | | | | | | | | |
|  | **Screening rate for those who performed a test, per 1000** | | | | | | | **Screening rate for all, per 1000** | | | | | | | | | | | |
|  | **2015** | **2016** | **2017** | **2018** | **2019** | **Linear trend** | | **2015** | **2016** | **2017** | | **2018** | | **2019** | | **Linear trend** | | | |
|  |  |  |  |  |  | **p-value** | **direction** |  |  |  |  |  |  |  |  | **p-value** | | **direction** | |
| **Overall** |  |  |  |  |  |  |  |  |  |  | |  | |  | |  | |  | |
| any screening modality | **66.69** | **77.11** | **92.17** | **78.38** | **108.37** | <0.1 | positive | **868.63** | **859.10** | **867.73** | | **857.59** | | **890.00** | | >0.1 | | N/A | |
| high sensitivity gFOBT (gFOBT) | 25.12 | 25.32 | 23.18 | 4.03 | 2.65 | <0.1 | negative | 251.65 | 196.92 | 150.91 | | 73.80 | | 52.11 | | 0.000239 | | negative | |
| FIT | 3.69 | 3.71 | 8.18 | 13.13 | 7.78 |  |  | 36.80 | 29.40 | 42.88 | | 59.70 | | 36.11 | |  |  |  |  |
| mt-sDNA | 1.34 | 4.29 | 10.69 | 16.12 | 52.02 | <0.1 | positive | 11.09 | 31.23 | 65.79 | | 105.56 | | 222.27 | | <0.05 | | positive | |
| screening Colonoscopy | 36.16 | 43.31 | 49.64 | 44.56 | 45.52 | >0.1 | N/A | 563.31 | 595.82 | 603.13 | | 613.24 | | 575.26 | | >0.1 | | N/A | |
| sigmoidoscopy | 0.37 | 0.47 | 0.48 | 0.55 | 0.40 | >0.1 | N/A | 5.79 | 5.73 | 5.02 | | 5.29 | | 4.24 | | <0.05 | | negative | |

| **Aged as of Dec 31st:**  **45 to 49** |  |  |  |  |  |  |  |  |  |  |  |  |  |  |
| --- | --- | --- | --- | --- | --- | --- | --- | --- | --- | --- | --- | --- | --- | --- |
| any screening modality | **19.30** | **23.03** | **21.91** | **18.43** | **17.76** | >0.1 | N/A | **841.39** | **818.73** | **809.76** | **806.05** | **811.65** | >0.1 | N/A |
| high sensitivity gFOBT (gFOBT) | 5.83 | 6.71 | 4.11 | 1.56 | 0.67 | <0.05 | negative | 185.80 | 149.04 | 82.49 | 40.56 | 20.85 | 0.00205 | negative |
| FIT | 1.41 | 1.39 | 1.44 | 2.16 | 2.28 |  |  | 46.83 | 30.21 | 31.14 | 41.30 | 42.42 |  |  |
| mt-sDNA | 0.00 | 0.05 | 0.00 | 0.00 | 0.00 | >0.1 | N/A | 0.00 | 1.01 | 0.84 | 0.74 | 0.00 | >0.1 | N/A |
| screening Colonoscopy | 11.66 | 14.67 | 16.27 | 14.56 | 14.81 | >0.1 | N/A | 596.68 | 631.42 | 688.55 | 717.55 | 741.91 | 0.00141 | positive |
| sigmoidoscopy | 0.40 | 0.20 | 0.10 | 0.15 | 0.00 | <0.05 | negative | 12.08 | 7.05 | 6.73 | 5.90 | 6.47 | >0.1 | N/A |
| **Aged as of Dec 31st: ≥76** |  |  |  |  |  |  |  |  |  |  |  |  |  |  |
| any screening modality | **47.36** | **55.45** | **61.63** | **57.37** | **59.68** | >0.1 | N/A | **808.58** | **774.54** | **771.53** | **765.26** | **777.10** | >0.1 | N/A |
| high sensitivity gFOBT (gFOBT) | 17.19 | 17.05 | 13.97 | 4.09 | 3.93 | <0.05 | negative | 243.19 | 189.40 | 144.88 | 87.22 | 71.66 | 0.00156 | negative |
| FIT | 10.45 | 10.40 | 13.05 | 14.09 | 13.83 |  |  | 133.51 | 98.18 | 88.31 | 88.42 | 80.59 |  |  |
| mt-sDNA | 0.94 | 3.14 | 5.76 | 7.72 | 16.75 | <0.05 | positive | 10.22 | 31.28 | 54.68 | 80.25 | 127.69 | <0.1 | positive |
| screening Colonoscopy | 18.33 | 23.82 | 27.63 | 30.29 | 24.14 | >0.1 | N/A | 410.08 | 443.53 | 470.14 | 495.92 | 485.79 | <0.05 | positive |
| sigmoidoscopy | 0.44 | 1.05 | 1.23 | 1.17 | 1.01 | >0.1 | N/A | 11.58 | 12.16 | 13.51 | 13.46 | 11.37 | >0.1 | N/A |
